# Endoscopic Femoropopliteal Bypass Surgery Without Groin Incision: A Feasibility Study on Cadavers with the Da Vinci Single Port System

**DOI:** 10.1016/j.ejvsvf.2026.04.005

**Published:** 2026-04-30

**Authors:** Rouven Berndt, E. Sebastian Debus, Hubert Stein, Mark Preuss, Glenn Stante, Fabien Thaveau

**Affiliations:** a Department of Vascular Medicine, University Heart & Vascular Centre Hamburg-Eppendorf, Hamburg, Germany; b Intuitive Surgical Inc., Sunnyvale, CA, USA; c Department of Vascular Surgery, University Hospital Clermont-Ferrand, Clermont-Ferrand, France

**Keywords:** da Vinci single port system, Endoscopic surgery, Fresh frozen cadaver, Peripheral bypass surgery, Robotic assisted surgery, Vascular surgery

## Abstract

**Introduction:**

Femoropopliteal bypass surgery (FPBS) is a standard surgical procedure but is still associated with complications, especially surgical site infections and groin seromas. This feasibility study aimed to investigate whether endoscopic, robot assisted surgery enabled FPBS with minimal surgical trauma and without a groin incision.

**Method:**

The da Vinci single port (SP) system (Intuitive Surgical, Sunnyvale, USA) was used to perform the novel technique of endoscopic FPBS on fresh frozen cadavers. For this novel technique, a 4 cm incision was made in the distal third of the medial thigh for supragenicular exposure of the proximal femoral artery. Thereafter, an end to side anastomosis between the common femoral artery and a polytetrafluoroethylene (PTFE) graft was performed endoscopically with the da Vinci SP system. Success of the procedure was defined by completion of the proximal bypass anastomosis and the avoidance of injuries to anatomic key structures. First evaluation of the technique was performed by assessment with a slightly modified Northwestern Objective Microanastomosis Assessment Tool (NOMAT) score.

**Results:**

Two proximal bypass anastomoses were successfully sutured without injury to the five pre-defined key anatomic structures. Skin to skin complete duration of the intervention including removal of the robotic system was 105 minutes and 92 minutes, respectively. The modified NOMAT score representing the efficiency of surgical handling and quality of the anastomosis was 29 of 45 and 37 of 45, respectively.

**Conclusion:**

The current study demonstrates that endoscopic, robot assisted FPBS was feasible within an acceptable timeframe and without injury to key anatomic structures. Further clinical development of endoscopic FPBS could enable minimally invasive bypass surgery including the advantages of conventional surgery but avoiding its limitations.

## Highlights

• First performance of endoscopic femoropopliteal bypass surgery (FPBS) in cadavers.
• Groin dissection and bypass surgery was performed with the da Vinci single port system.
• Moderately trained robotic surgeons were able to create FPBS in under 120 minutes.

## INTRODUCTION

Femoropopliteal bypass surgery (FPBS) is the gold standard for patients with peripheral arterial disease who have undergone unsuccessful endovascular therapy or who suffer from chronic limb threatening ischaemia (CLTI).^1^^,^^2^ Although first described over 75 years ago by Jean Kunlin, its fundamental advantages and limitations remain unchanged.^1^^,^^2^ The BEST-CLI trial demonstrated durable long term patency rates and acceptable limb salvage outcomes in patients with CLTI.^1^ However, these benefits come at the cost of significant surgical trauma and a high incidence of groin surgical site infections, reported to be as high as 44% in some series.^1^, ^2^, ^3^

Several surgical disciplines have established techniques to reduce surgical trauma while preserving the benefits of conventional approaches; arthroscopy in orthopaedic surgery and robot assisted visceral surgery are prominent examples.^4^^,^^5^ In contrast, minimally invasive vascular surgery has largely evolved toward an “endovascular first” paradigm, representing a distinct treatment strategy. However, minimally invasive surgical alternatives to FPBS remain poorly explored, particularly in the context of robotic and endoscopic techniques. Therefore, this study evaluated the feasibility of performing FPBS as an endoscopic procedure using the da Vinci single port (SP) system (Intuitive Surgical, Sunnyvale, USA).

### Study design

The study was conducted as a pre-clinical, feasibility study designed for usability and technical validation of the da Vinci SP system for FPBS. All experiments were performed on fresh frozen cadavers (FFC). The primary endpoint was technical feasibility of the procedure. Secondary endpoints included the first qualitative evaluation of the novel technique and operating procedure time.

## METHOD

### Ethics

After approval of the local ethical committee of the University of Strasbourg, all procedures were performed at the Institut de Recherche contre les Cancers de l'Appareil Digestif (IRCAD) (Strasbourg, France) certified for medical research and development (DIN EN ISO 9001:2015).

### Development of the surgical technique

**Figure 1:**
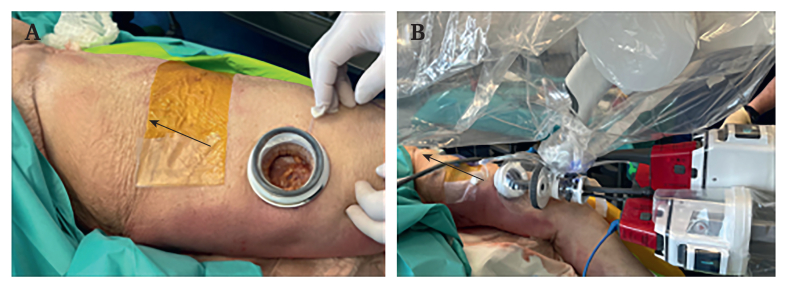
Photographic images demonstrating (A) introduction of the port system through a conventional medial supragenicular exposure; (B) endoscopic dissection of the groin began after docking the da Vinci single port system. Black arrow = direction of the dissection.

All surgical experiments (*n* = 2) were performed on FFC with the da Vinci SP system, which is a robotic surgical platform enabling laparo-endoscopic surgery through a single, small incision. Surgery was performed by two robot trained vascular surgeons (M.P., F.T.) with an experience level ranging from fewer than ten cases of robotic assisted surgery (M.P.) to more than 50 cases (F.T.). Three instruments and an endoscope were controlled by the surgeons through a single port, which was applied through conventional medial supragenicular exposure (Fig. 1A).

The cadaver was placed in a slightly supine position with the leg externally rotated and the knee flexed to 30° (Fig. 1B). A 4 cm incision was made in the distal third of the medial thigh along the anterior border of the sartorius muscle (supragenicular exposure).^6^ The fascia lata over the sartorius muscle was incised for later conventional preparation of the proximal popliteal artery and distal bypass grafting.

The SP access port was then placed under the subcutaneous tissues, carbon dioxide (CO_2_) insufflation was started at 8 mmHg, and the da Vinci SP system was introduced. In general, fenestrated bipolar forceps, monopolar curved scissors, and a Cadiere forceps were used for the endoscopic surgical preparation with the da Vinci SP system. Clipping of branches was performed with disposable Hem-O-Lok ligating clips (Teleflex Inc., Morrisville, USA).

**Figure 2:**
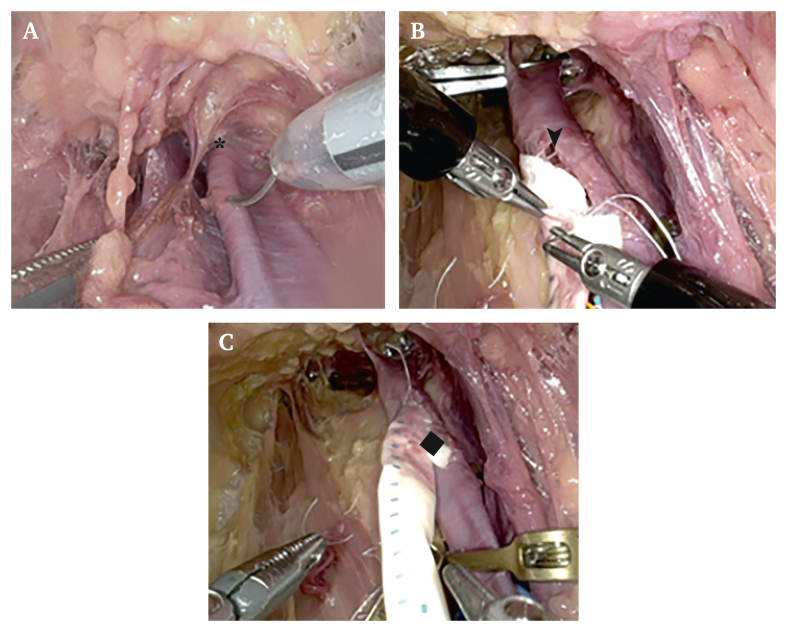
Photographic images demonstrating **(**A) endoscopic dissection of the groin with the da Vinci single port system and preparation of the superficial femoral artery (black asterisk); (B) endoscopic suturing of the proximal bypass anastomosis (black arrow); (C) completed endoscopic sutured bypass anastomosis of the common femoral artery (black diamond).

Initial preparation was performed on the surface of the fascia lata, and subcutaneous tissues were carefully dissected following the vascular route in the direction of the groin (Fig. 1B and Video). The CO_2_ insufflation supported the surgical preparation by tissue suppression. Approximately 3 – 4 cm before the bifurcation, the fascia lata was incised and dissection of the femoral bifurcation began (Video). The incision was further deepened through the fascia between the vastus medialis and adductor longus muscles.^6^ In contrast to open surgery, the sartorius was mobilised medially to gain access to the vascular route (Video). The common femoral (CFA), profunda femoris (PFA), and superficial femoral arteries (SFA) were carefully dissected and encircled for control with vessel loops (Fig. 2A). Debakey robotic bulldog clamps (DUFNER instruments, Tuttlingen, Germany) were used for cross clamping. An end to side anastomosis with a polytetrafluoroethylene (PTFE) graft (W. L. Gore & Associates, Inc., Newark, USA) was performed robotically with two large needle drivers (Fig. 2B and 2C). For suturing, two single armed CV-6 Gore-Tex sutures (W. L. Gore & Associates, Inc.), cut to 10 cm, were used. At the end of the proximal anastomosis, the graft was pulled through from the proximal popliteal access using the conventional surgical technique and anatomic route. After removal of the da Vinci SP system, the initial incision of the SP port was used to dissect the proximal popliteal artery to perform a conventional distal bypass anastomosis.

Supplementary video related to this article can be found at https://doi.org/10.1016/j.ejvsvf.2026.04.005.

The following is/are the supplementary data related to this article:

Video 1 Endoscopic access to the femoral bifurcation with the da Vinci single port system and performance of the proximal bypass anastomoses with a polytetrafluoroethylene graft.

### Evaluation of the surgical technique

Success of both procedures and the underlying technique was defined *a priori* by endoscopic completion of the proximal femoral bypass anastomoses and prevention of damage to key anatomic structures: major arterial and venous vessels, perforation of skin and subcutis, the femoral nerve, sartorius muscle, and inguinal ligament. Measurement of the surgical procedure duration began after the robot system was docked and ended with the knot and finalisation of the bypass anastomosis. First evaluation of the novel technique was assessed by applying a slightly modified, objective assessment scale (i.e., the Northwestern Objective Microanastomosis Assessment Tool [NOMAT]), which was blindly graded by a senior vascular surgeon.^7^ Additionally, all surgical cavities were opened and the anastomoses were examined directly by the surgeons. The modified scale of the NOMAT score focused on the efficiency of surgical handling and quality of anastomosis (items IV – XII, range 0 – 45) and was assessed by video recording in a blinded manner.

## RESULTS

Both experiments were performed without technical complications or injuries to the five pre-defined key anatomic structures. The robotic system docking times were 12 and 14 minutes, respectively. Dissection of the groin took 22 minutes for the first experiment and 26 minutes for the second. Completion of the proximal, robot assisted bypass anastomosis was performed in 46 minutes and 17 minutes; skin to skin complete duration of the intervention including the distal anastomosis and removal of the robotic system was 157 and 142 minutes, respectively. The modified NOMAT score including the items for the efficiency of the surgical handling and the quality of the anastomosis was 29 of 45 and 37 of 45, respectively.

## DISCUSSION

In the current study, the authors demonstrated the feasibility of a complete dissection of the CFA, PFA, and SFA, and the completion of two CFA bypass anastomoses via an endoscopic, robot assisted technique with the da Vinci SP system. Endoscopic surgery was performed within an acceptable time by moderately experienced robot vascular surgeons, without any major injuries of the anatomic key structures. Interestingly, the acceptable time frame of both procedures and the increasing NOMAT score suggest that both vascular surgeons had little difficulty adapting to the new procedure and endoscopic technique, which had previously only been planned theoretically. In the second experiment, 82% of the possible points were recorded, which the authors considered an acceptable result for a novel procedure. Accordingly, the performance of endoscopic FPBS without groin incision might also be feasible in a clinical setting.

The general advantages of robot assisted surgery over conventional surgery have been described for general surgery, urology, and gynaecology, and included less surgical trauma, less wound healing disorder, and shorter in hospital stay, as well as technological advances such as high precision movements of the surgical instruments and three dimensional visualisation.^8^^,^^9^ Due to the increasing use of endovascular techniques, robotic surgery has so far hardly played a role in vascular surgery, although it has been a fairly natural successor to laparoscopy. Fundamental problems include the lack of haptics in the performance, technical skills leading to a longer learning curve, although shorter than laparoscopy for vascular anastomoses, problems in bleeding control, and high material costs.^8^^,^^10^^,^^11^ However, developments over time and determination of tertiary centre surgical teams have made it possible to show that robotic surgery can push the limits of laparoscopy and perform complex vascular procedures such as aorto-iliac surgery.^12^^,^^13^ However, further significant developments in robotic surgery will also include haptic feedback for the surgeon as well as smaller systems for microsurgery, and might push the use of robotic systems towards a more common surgical approach, even in vascular surgery.^8^^,^^12^

Although, FPBS has been a well described procedure in patients with CLTI and or peripheral arterial disease for almost eight decades, there have been challenges and pitfalls, and relatively stable morbidity and mortality rates over the years.^1^^,^^2^ Complications have mostly been related to major bleeding and post-operative wound complications of the dissected groin. Surgical site infections and seromas of the dissected groin have particularly represented a significant number of complications after vascular procedures and the incidence has ranged 3–44%.^3^^,^^14^ In contrast, major advantages of conventional FPBS arise from the durability and long term patency of the revascularisation over endovascular procedures.^1^ Therefore, groin exploration without incision might be a significant advance in vascular surgical techniques and might balance a significant proportion of the disadvantages of FPBS.^14^ The potential evolution of conventional bypass surgery into an endoscopic operation could therefore be comparable with the further advance of conventional surgical techniques in other disciplines (e.g., arthroscopy in orthopaedic surgery).^5^ The only previously available robotic systems were multiport systems, which were not specifically designed to perform this type of surgery on the femoral bifurcation. Consequently, the use of the da Vinci SP system for the precise performance of anastomoses and preparation in confined cavities is estimated by the authors as a potential major advantage in the further development of FPBS.^8^^,^^13^^,^^15^ Although only PTFE grafts were employed in the current experiments, the da Vinci SP system is also suitable for performing microsurgical vascular anastomoses with autologous bypasses. Furthermore, the use of new haptic systems and microsurgical instruments will continue to change robotic surgery.^12^^,^^16^

In the current experiment, only the access to the proximal popliteal artery was dissected. However, the approach also appears suitable for bypass procedures on the distal popliteal artery or the crural vessels, with additional surgical access on the lower leg. Nevertheless, the advantage of a non-dissected groin would remain.

Naturally, this early feasibility study is preliminary and has several limitations. Firstly, all surgical experiments were performed in a FFC and therefore no statement in terms of sufficiency of the anastomosis or bleeding due to vessel injury could be made. However, a modified NOMAT score > 50% makes it highly likely that the anastomoses might have been sufficient even under perfusion. Secondly, CO_2_ insufflation and preparation created a subcutaneous tunnel. Accordingly, further clinical evaluation is needed to investigate post-operative wound healing and potential complications (e.g., lymphatic leakage).

### Conclusions

This study has demonstrated that endoscopic, robot assisted FPBS was successfully performed within an acceptable timeframe and without injury to key anatomic structures in a cadaver model. Clinical development of this endoscopic robot assisted technique could lead to a minimally invasive approach for FPBS, including all the advantages of conventional surgery without several limitations. The next steps include a first clinical safety concept and a physician sponsored and institutionally sanctioned initial clinical study of the da Vinci SP system for FPBS.

## Funding

No funding was received for these experiments. All experiments were performed with the support of the Institut de Recherche contre les Cancers de l'Appareil Digestif (IRCAD) (Strasbourg, France).

## CONFLICT OF INTEREST

The robotic procedures were performed with the da Vinci SP system provided by Intuitive Surgical for the purpose of clinical research to the Institut de Recherche contre les Cancers de l'Appareil Digestif (IRCAD) (Strasbourg, France). H.S. and G.S. are currently employees of Intuitive Surgical.
